# Polymeric microcarriers for minimally-invasive cell delivery

**DOI:** 10.3389/fbioe.2023.1076179

**Published:** 2023-01-26

**Authors:** Chunyan Duan, Mingjia Yu, Changji Hu, Hongying Xia, Ranjith Kumar Kankala

**Affiliations:** ^1^ School of New Energy and Environmental Protection Engineering, Foshan Polytechnic, Foshan, China; ^2^ Fujian Provincial Key Laboratory of Biochemical Technology, Institute of Biomaterials and Tissue Engineering, College of Chemical Engineering, Huaqiao University, Xiamen, China

**Keywords:** tissue engineering, minimally-invasive, microfluidic technology, polymeric carriers, injectable architectures

## Abstract

Tissue engineering (TE) aims at restoring tissue defects by applying the three-dimensional (3D) biomimetic pre-formed scaffolds to restore, maintain, and enhance tissue growth. Broadly speaking, this approach has created a potential impact in anticipating organ-building, which could reduce the need for organ replacement therapy. However, the implantation of such cell-laden biomimetic constructs based on substantial open surgeries often results in severe inflammatory reactions at the incision site, leading to the generation of a harsh adverse environment where cell survival is low. To overcome such limitations, micro-sized injectable modularized units based on various biofabrication approaches as ideal delivery vehicles for cells and various growth factors have garnered compelling interest owing to their minimally-invasive nature, ease of packing cells, and improved cell retention efficacy. Several advancements have been made in fabricating various 3D biomimetic microscale carriers for cell delivery applications. In this review, we explicitly discuss the progress of the microscale cell carriers that potentially pushed the borders of TE, highlighting their design, ability to deliver cells and substantial tissue growth *in situ* and *in vivo* from different viewpoints of materials chemistry and biology. Finally, we summarize the perspectives highlighting current challenges and expanding opportunities of these innovative carriers.

## 1 Introduction

Despite the sophisticated surgical reconstruction procedures costing billions of dollars, end-stage organ failure or tissue loss is bothering each year, resulting in millions of deaths. In addition, the shortage of donors for organ replacement healing worsens the problem (Langer and Vacanti, 1993). Although the life span is prolonged to some extent, the currently available solutions remain imperfect in treating these tissue defects. Moreover, these surgical reconstruction procedures using various techniques, such as mechanical devices, often lead to long-term problems and, eventually, deterioration (Santana et al., 2020). Over the decades, several efforts in developing diverse, innovative technologies for next-generation medical treatments have continued to rise.

Recently, tissue engineering (TE) and regenerative medicine (RM) fields have garnered captivating interest owing to their promising potential for repairing tissue defects ensuing from chronic infections and aging (Langer and Vacanti, 1993). Conceptually, this innovative biomedical field assimilates several disciplines, including chemistry, engineering, biology, and material science, to fabricate arbitrary-sized three-dimensional (3D) biomimetic tissue constructs with enriched performance. These fabricated systems can reinstate the structure and function of the malfunctioned tissues, considering the homeostasis regulation, physiochemical factors, and biochemical cues required for tissue growth (Kankala et al., 2017; Kankala et al., 2018a). Moreover, the fabrication of such complex physiological systems requires accumulated knowledge on the deepened understanding of tissue-specific microenvironments as well as various dynamic structural organizations, involving cell-matrix-based biophysical and biochemical interactions, as well as cell-cell crosstalk using the intercellular components (integrin, selectin, and other cell adhesion molecules and extracellular matrix, ECM, proteins), among others (Li et al., 2015; Jiang et al., 2016). Intriguingly, this area of research has already shown promising outcomes acting against diabetes, cancer, skin burns, osteoarthritis, cardiovascular conditions, congenital disabilities, injured tissue sections, and tumor resections (Leijten et al., 2016).

In past decade, efforts have been dedicated to develop engineered implantable scaffolds mimicking the anatomical and physiological aspects of the tissue microenvironment, ranging from the organ stage to the tissue and cellular levels (Liu et al., 2019). Along this line, several scaffolding systems that could emulate the native counterparts have been fabricated, such as photo-cross-linkable hydrogels, biodegradable porous scaffolds, and nano/microfibrous biocompatible materials (Martin et al., 2004; Asakawa et al., 2010). Considering the highly organized complexity in both areas of cell surfaces and intracellularly, further advancements on the nanoscale have also been made by researchers in search of innovative nano-sized components to augment the intrinsic performance of large-sized scaffolds (Kankala et al., 2018a). Compared to the implantable bulk scaffolds, these 3D scaffolding systems combining the micro- and nano-sized components play pivotal roles in repairing the malfunctioned tissues and offering efficient control over the microenvironment for cell and tissue growth (Ferrari et al., 1998; Kankala et al., 2018a). These injectable biomaterials present a new era of minimally-invasive therapeutics, representing the delivery of biologics, drugs, and other bioactives. To solve tissue defects or fill the irregularities in the tissues, the micro-sized carriers can encapsulate cells in their interiors and deliver them appropriately in the region of interest towards improved cell proliferation and subsequent tissue growth. Various kinds of microcarriers for cell delivery have been fabricated, depending on porosity (porous and non-porous/solid microcarriers) and texture (rigid and soft), among others. Compared to bulk scaffolds, these microcarriers offer several advantages, such as enhanced cell encapsulation and retention efficiencies, improved cell proliferation in the interiors, and minimally invasiveness, among others (Van Wezel, 1967; Khademhosseini et al., 2006; Jiang et al., 2016). Although the design of microcarriers for cell delivery is successful, it should be noted that several factors, such as porosity, cell retention efficacy, and administration route, play predominant roles in the success of these minimally-invasive cell carriers for TE (Khademhosseini et al., 2006). These modular units for minimally-invasive delivery of cells have been developed for various organs in the body, such as hepatic (Liu et al., 2014), bone (Ligorio et al., 2019), chondral (Malda et al., 2003), muscle (Kankala et al., 2019) skin (Gualeni et al., 2018), and neural (Jeon et al., 2021) among others (Langer and Vacanti, 1993; Hollister, 2005; Khademhosseini et al., 2006; Bhatia and Ingber, 2014; Wang et al., 2018). In addition to the regeneration of various tissues, these modular units for TE can be applied to repair various tissues, including congenital disabilities, deep-cut injuries, and areas of tumor resections, among others.

**FIGURE 1:**
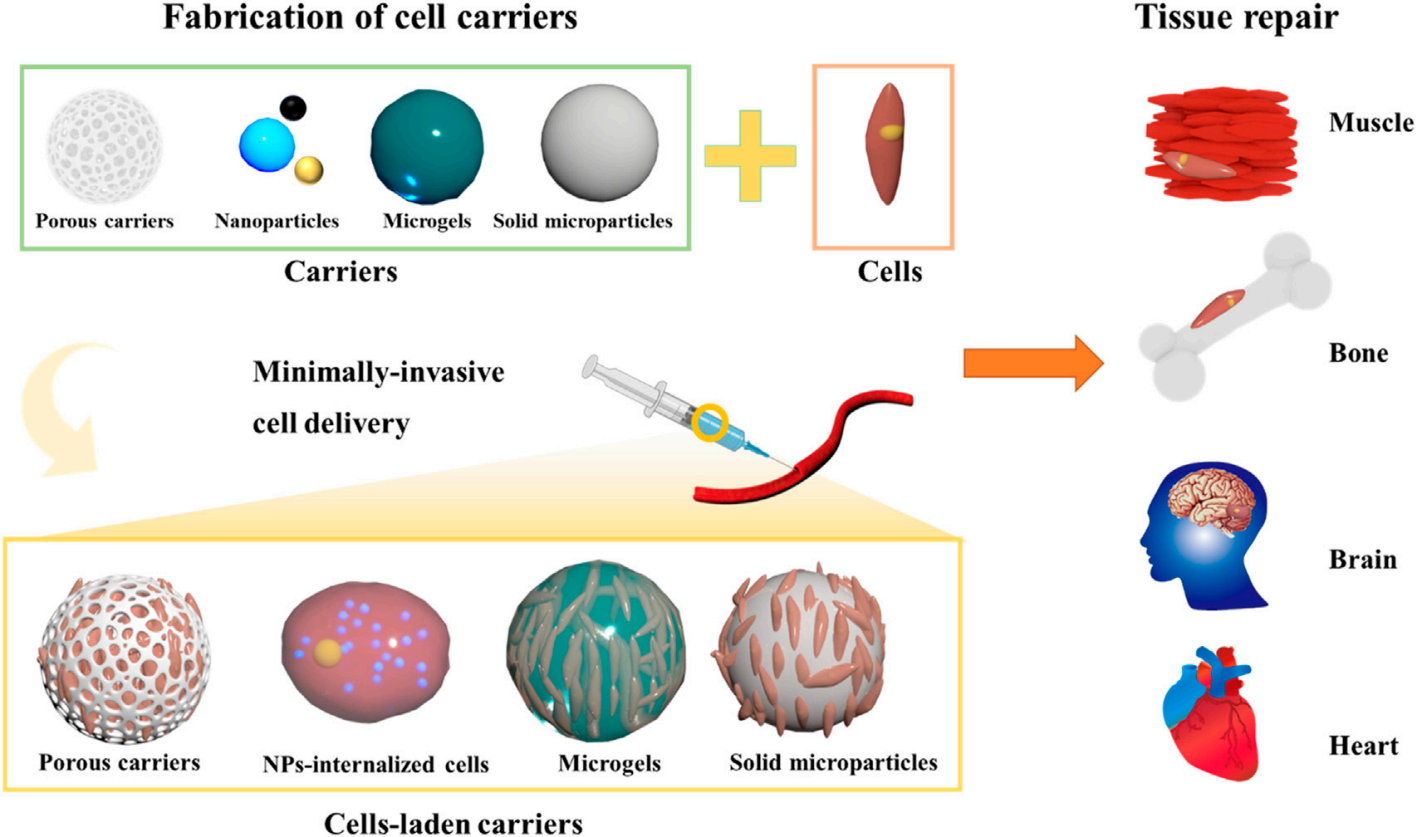
Schematic illustrating several kinds of 3D microcarriers for cell delivery applications.

Although reviews have been published on exploring the potential of TE, (Khademhosseini et al., 2006; Bhatia and Ingber, 2014; Li et al., 2015; Kankala et al., 2018b; Li et al., 2018) only a few are focused on the utilization of functional micro- as well as nano-sized constructs as minimally-invasive cell delivery vehicles towards repairing the malfunctioned tissues either through facilitating the natural ECM-like environment and drug delivery characteristics. Motivated by these considerations, in this compilation, herein we give a comprehensive overview of the microarchitectures for minimally-invasive cell delivery towards the growth of various tissues and substantial enrichment of molecular cues in guiding vascularization and nerve innervation processes. Initially, we emphasize the significance and classification of microarchitectures towards cell delivery, highlighting their importance compared to the bulk scaffolds and non-scaffolds-based designs for TE (Figure 1). Then, we highlight various engineering strategies utilized to fabricate various carriers for cell delivery applications. Further, we emphasize various micro-sized carriers for cell delivery and factors affecting their performance efficiency. Finally, a note on the applicability of these carriers to different engineering tissues is emphasized.

## 2 Significance of microarchitectures

Over the past few decades, tremendous efforts have resulted in fabricating various artificial tissue constructs using biomaterials based on organic and inorganic-based materials for tissue regeneration (Braccini et al., 2020). In addition to tissue growth, these biomaterials can be applied for vascularization and nerve innervation, improving the tissue repair efficiency of these implanted scaffolds (Langer and Vacanti, 1993). To mimic the natural tissues, further advancements in various tremendous technologies have been evidenced in the generation of highly organized artificial 3D constructs composed of different cell types, ECM, and numerous signaling cues (Liu et al., 2019). Along this line, various scaffold-free and scaffold-based designs have been utilized for replicating the natural constituents of human tissues (Song et al., 2017). On the one hand, scaffold-free cell-rich architectures have been developed by generating 3D cell aggregates for TE (Lee et al., 2007). Notably, these 3D cell aggregates replicate the native tissue environment, in terms of hypoxia, pH, protein expressions, cell-cell interactions, and growth factor profiling, among others (Lu and Stenzel, 2018; Zanoni et al., 2019). These cellular blocks are also estimated to be programmed hierarchical assemblies with a precise design toward organ-like structures. It should be noted that the assembly of cell aggregates reduces the possible risk factors of cell-based therapies and guide cellular differentiation (Kankala et al., 2018b). Despite the advantages of replicating the natural intricacies, these cell-based aggregates are often loosely bound, lacking the critical components of cell-ECM interactions (integrins, cadherins, and selectins-ECM proteins), heterogeneity in sizes, and growth trends (Kankala et al., 2019). Moreover, the cells in the interior of the spheroids may suffer from a deprived survival rate due to hypoxia conditions and lack the vascularization, limiting their applicability in TE. In addition, the hierarchical assembly of the cells as tissue organization and biomimetic designs may certainly limit somatic mutations (Khademhosseini and Langer, 2016). On the other hand, scaffold-based architectures have been developed to address the limitations mentioned above of scaffold-free architectures (Hollister, 2005). These scaffolding materials from biodegradable polymeric materials support bulk materials for improving adhesion and substantial tissue growth (Choi et al., 2012). Various solid architectures, including photo-cross-linkable hydrogels, biodegradable scaffolding systems, and nano/microfibrous biocompatible constructs, have been developed (Martin et al., 2004; Asakawa et al., 2010; Choi et al., 2012). The regulated interplay and the intricately controlled crosstalk between the materials and the biological components played substantial roles in TE and RM toward tissue growth (Khademhosseini and Langer, 2016). However, various key attributes, such as growth factors and their precise actions, would play the predominant role in cell proliferation, requiring them in the engineered constructs in *ex-vivo* and *in vivo* (Kankala et al., 2018a; Braccini et al., 2020). Despite the success, the large-sized scaffolds require sophisticated, highly invasive surgical procedures, leaving a scar (Wei et al., 2018). These surgical incisions result in a substantial generation of inflammatory reactions resulting in a harsh environment, where the survival of cells in the implanted scaffolds remains low (Kankala et al., 2019).

Various biofabrication approaches have been developed to generate micron-sized templates, which could be convenient for minimally-invasive delivery with improved cell adhesion and tissue growth trends to address the limitations (Wei et al., 2018). Indeed, these micron-sized carriers, with an average size ranging from 1 to 1,000 μm, offer various advantages of widespread encapsulation and carrying abilities, biodegradability, and biocompatibility for various biomedical applications (Li et al., 2015; Jiang et al., 2016; Khademhosseini and Langer, 2016; Kankala et al., 2018b; Imai et al., 2018; Li et al., 2018; Liu et al., 2019; Santana et al., 2020). Since, ever the first report on encapsulation of mammal cells in diethyl aminoethyl (DEAE)-Sephadex A50 from Van Wezel (1967), several efforts resulted in diverse varieties of microengineered 3D architectures for encapsulating cells and their subsequent *ex-vivo* expansion (Khademhosseini et al., 2006; Li et al., 2015; Jiang et al., 2016; Santana et al., 2020). These 3D structures offer several benefits, such as high surface area, efficient monitoring, and a convenient supply of nutrients (Li et al., 2018). Despite the advantages, several other notable factors depend on the successful adhesion and growth of cells, such as chemical nature and compatibility, physical characteristics, surface properties, and porosity (Hollister, 2005). Indeed, material compatibility is often influenced by the applied substrates’ chemical composition. In most instances, biocompatible polymers from natural (chitosan, dextran, gelatin, and alginic acid) (Bae et al., 2006; Huang et al., 2018; Chen et al., 2015) and synthetic [poly(lactic-*co*-glycolic acid, PLGA, polyurethane, polylactic acid, PLA, polycaprolactone, PCL, polyhydroxyalkanoate (PHA), polystyrene, polyhydroxylethyl methacrylate, PHEMA, and polyacrylamide] (Liu et al., 2011; Kim et al., 2016; Kankala et al., 2019) origins are often applied to generate 3D microarchitectures. The critical morphological characteristics (size and shape) play significant roles in designing these architectures, facilitating improved encapsulation, convenient administration, and delivery efficiencies (Li et al., 2018). In general, microcarriers with a spherical shape and an average size of 100–500 μm would facilitate the encapsulation of numerous cells, avoiding cell necrosis in their interiors (Hong et al., 2005; Wei et al., 2018). In addition to chemical nature and physical characteristics, surface texture and porosity are other significant features that play crucial roles in the encapsulation efficiency of diverse cell types (Wei et al., 2018). Initially, the fabricated biocompatible solid microspheres with a rough surface and tiny pores have shown improved adhesion efficiency on the surface compared to the microspheres with smooth surfaces due to the improved and non-slippery interactions between the cells and microcarriers (Matsushita, 2020). Although the rough surfaces provide improved adhesion of cells, these solid microcarriers suffer from a significant limitation of low encapsulation yields due to less surface area (Choi et al., 2012). To overcome this limitation, several biofabrication approaches have been explored in the generation of porous microspheres (PMs) and their highly open prototype with controlled structure and porosity. These microarchitectures with high surface area, heterogeneous porosity in the range of 10–100 μm, and interconnected windows subsequently facilitate a series of events, such as the adhesion of cells initially on the surface and improved migration to the interiors through the pores (Wei et al., 2018). In addition, the abundant porosity of the biocompatible carriers enables the interchange of gases and nutrients, facilitating improved viability and proliferation efficiencies of the harbored cells in the interiors (Choi et al., 2012). The material characteristics and the cellularized secretions can generate and mimic the ECM-like environment (Hollister, 2005). Nonetheless, several biochemical cues and the desired architectures are required as a significant prerequisite for TE to substantially control the microenvironment. Considerably, the combination of polymers from both natural and synthetic origins can be applied to improving the compatibility, increasing the biochemical cues, and varying the mechanical properties of the 3D microcarriers (Pradhan et al., 2017).

## 3 Classification of cell carriers

Broadly speaking, the cell delivery microcarriers can be categorized into two major classes based on the arrangement of cells and the size constraints, such as cells-encapsulated microcarriers and cells internalized with micro/nanocarriers (Hafeman et al., 2008; Xu et al., 2019). The cells-laden microcarriers are often based on polymeric microarchitectures, referred to as the large-sized carriers in which the cells can be accommodated. On the one hand, considering the structural attributes, these micro-sized carriers can be further classified into solid (non-porous/porous) microparticles and liquid-rich micro-sized gels (Oyama et al., 2014; Wei et al., 2018). On the other hand, the sub-micro-sized particles can be utilized to improve the delivery of cells to the target tissue for tissue repair. In this section, we present an overview of these cell carriers (Table 1), highlighting the emphasis on encapsulation and delivery efficacies of cells from their interiors to the target organs for tissue repair.

**TABLE 1:** Summary of various cell-laden carriers for minimally-invasive delivery of cells for tissue repair and regeneration purposes.

Design	Material	Micro-engineering strategy	Delivered cell type	Size	Targeted site	Outcome	References
Solidified PMs	PLGA	Microfluidics	Skeletal myoblasts	280–370 μm	Skeletal muscle	These microcarriers improved cell retention and vascularization and partial myoblast differentiation in mice	Kankala et al. (2019)
	Alginate-gelatin microspheres	Electrospray	Adipose-derived stem cells (ADSCs)	360 μm	Knee cartilage	Microspheres increased the viability of ADSCs and supported their proliferation and deposition of cartilage matrix	Liao et al. (2022)
	Collagen-Poly-lactide (PLA)	Emulsion-solvent evaporation	Chondrocyte	180–280 μm	Cartilage	The larger amount of collagen on these PLA microspheres could attach, proliferate and spread chondrocytes	Hong et al. (2005)
	Polycaprolactone (PCL)	Molding	Neural progenitor cells	244–601 μm	Brain	PC-12 cells attached to microspheres were populated within their macropores, applicable for neuron TE.	Kim et al. (2016)
	Polyhydroxyalkanoate (PHA)	Double emulsification	human bone marrow mesenchymal stem cells (BMMSCs)	300–360 μm	Defect tissues	PHA OPMs protected cells against stresses during injection, allowing more living cells to proliferate and migrate to damaged tissues	Wei et al. (2018)
	Calcium-alginate	Freeze-drying	Osteoblasts	5 mm * 5 mm (2D)	Bone	This system preserved the cell proliferation and upregulated bone-related gene expression towards skeletal defects	Chen et al. (2015)
	Star-shaped PLA	Self-assembly	Chondrocytes	60 μm	Knee repair	The nanofibrous hollow microspheres achieved better cartilage repair than chondrocytes-alone	Liu et al. (2011)
	Gelatin methacryloyl (GelMA)-alginate core-shell microcapsules	Co-axial electrostatic microdroplet	HDPSCs and HUVECs	∼359 μm	Tooth	Microvessel formation and pulp-like tissue regeneration occurred in the co-culture group toward endodontic regeneration	Liang et al. (2022)
	PCL–gelatin	Electrospinning and electrospraying	Rat BMMSCs	125–200 μm	Bone marrow	The microspheres improved the viability and maintenance of stem cells for cell therapy	Boda et al. (2018)
	Methacrylated hyaluronic acid (HA) and N-vinylpyrrolidone	Photopolymerization	Bovine articular chondrocytes	2.5–2.9 mm	Repairing tissue defects	HA hydrogel beads could be used as injectable cell delivery vehicles	Bae et al. (2006)
	PCL	Isolated particle-melting method and melt-molding particulate-leaching	Chondrocytes	400–550 μm	Cell delivery	The PCL microscaffolds showed biocompatibility and infiltration of cells for cell delivery applications	Lim et al. (2009)
	Sodium alginate (SA)/TOCNF and *β*-Tricalcium phosphate	Droplet extrusion- crosslinking technique	MC3T3-E1	1.25 mm	Bone	The prepared microspheres showed significantly better bone formation in a rabbit model than in the control group	Ho et al. (2020)
	Silk fibroin/gelatin (SF/G)	Self-assembly	Rat MSCs	300–400 μm	Bone	SF/G microcarriers supported the adhesion of rat MSCs with high efficiency under dynamic culture	Luetchford et al. (2020)
	Acrylic acid onto plasma-treated poly(ethylene terephthalate)	Graft polymerization	Smooth muscle cells	—	Smooth muscle cells	Collagen-immobilized surfaces increased the surface area and subsequent substrate for cell seeding	Gupta et al. (2002)
	Flexible wood membrane	Chemical treatment	HEK293 cells	—	Surgical practices	The extracted material based on flexible wood could be used as a 3D bioscaffold	Song et al. (2017)
	Alginate	Chemical cross-linking	HepG2	90–900 μm	Cell delivery	These scaffolds supported the expansion of HepG2 and maintained the albumin secretion function	Li et al. (2014)
	Chitosan	Emulsion-based thermal-induced phase separation	Hepatocyte	150 μm	3D cell culture	The biocompatibility and porous structure attributes resulted in the high performance of hepatocyte culture.	Huang et al. (2018)
Microgels	Magnetic microcryogels based on gelatin and Poly(ethylene glycol) diacrylate (PEGDA)	Cryogelation and micro-molding	HepaRG	400 μm	Hepatic	The robust, controllable, and magnetic resonance imaging (MRI) traceable magnetic microtissues are provided to solve multiple critical issues in TE and RM.	Liu et al. (2014)
	Graphene oxide (GO)-β-sheet forming self-assembling peptide hydrogels	Hybrid injectable 3D scaffolds	Nucleus pulposus cells	—	Intervertebral disc (IVD)	These hybrid hydrogels promoted high cell viability and retained cell metabolic activity for IVD degeneration	Ligorio et al. (2019)
	Microscale alginate beads- thermosensitive hydrogel	Electrospray	MSCs	>200 μm	Skin	The arrangement of collagen fibrils and high angiogenesis confirmed the wound-healing process of the hydrogel	Nilforoushzadeh et al. (2020)
	Modified gelatin matrix with PLA-co-trimethyl carbonate [P(DLLA-co-TMC)]	UV Crosslinking	Embryonic stem cells (ESCs)	Gels	Spinal cord injury	The ESCs-loaded composite hydrogels are identified to enhance tissue regeneration and motor function significantly	Wang et al. (2018)
	Quaternized *β*-Chitin (QC) and oxidized dextran (OD)	Schiff base reaction	NIH-3T3 and mouse BMMSCs	Gels	3D culture	These gels act as antibacterial vehicles for delivering cells toward RM, drug/gene/cell delivery, and cell therapy	Xu et al. (2019)
	Poly(lactide-co-glycolide)-b-poly(ethylene glycol)-b-poly(lactide-co-glycolide) and clay nanosheets	Self-assembly	L929 cells	Gels	—	These gels with appropriate compatibility features and textural attributes could be used as injectable cell delivery carriers	Oyama et al. (2014)
	Keratin allyl thioether biopolymer	Photo-crosslinking	hMSCs	Gels	Bone and cartilage	The keratin allyl thioether hydrogel with controllable degradation could act as a viable matrix for encapsulation and delivery of stem cells for tissue repair	Barati et al. (2017)
	PLGA-chitosan/PLGA-alginate	Self-assembly homogenization	Human umbilical cord mesenchymal stem cells (hUCMSCs)	Gels	TE and drug release	These biodegradable colloidal gels could act as injectable scaffolds for tissue repair	Wang et al. (2011)
Cells internalized with micro/nanocarriers	Superparamagnetic iron oxide nanoparticles	Poly-L-lysine- surface modified	Human nasal turbinate stem cells	Iron oxide: 15–30 nm	Brain	The intranasal administration of cells internalized with nanocarriers could effectively treat CNS disorders	Jeon et al. (2021)

### 3.1 Solid (non-porous/porous) microcarriers

By employing different microfabrication approaches, diverse varieties of microcarriers have been generated towards engineering various tissue defects, such as bone (Chen et al., 2015), muscle (Kankala et al., 2019), dental (Liang et al., 2022), cartilage (Hong et al., 2005), brain (Kim et al., 2016), and hepatocytes (Huang et al., 2018) among others (Bae et al., 2006). Considering the porosity, these solid microcarriers can be classified into non-porous and porous carriers. The former type possesses no substantial pores on their surfaces but with micro-sized pores. In contrast, the latter carrier contains highly-open pores with interconnecting windows. These microcarriers present improved adhesion and subsequent carrying ability of diverse cell types, as well as cell proliferation and growth abilities on their surfaces and interiors (Almería et al., 2010). Comparatively, the open porous microcarriers facilitate more viability of cells in terms of encapsulation and delivery efficacies compared to the non-porous type. Nevertheless, the non-porous microcarriers with tiny pores intuitively allow the cells to adhere to their surfaces, facilitating improved delivery efficacy (Li et al., 2014; Akamatsu et al., 2018). These microcarriers also offer advantages, such as ease of fabrication and validation, biodegradability, biocompatibility, and cost-effectiveness (Choi et al., 2012).

**FIGURE 2:**
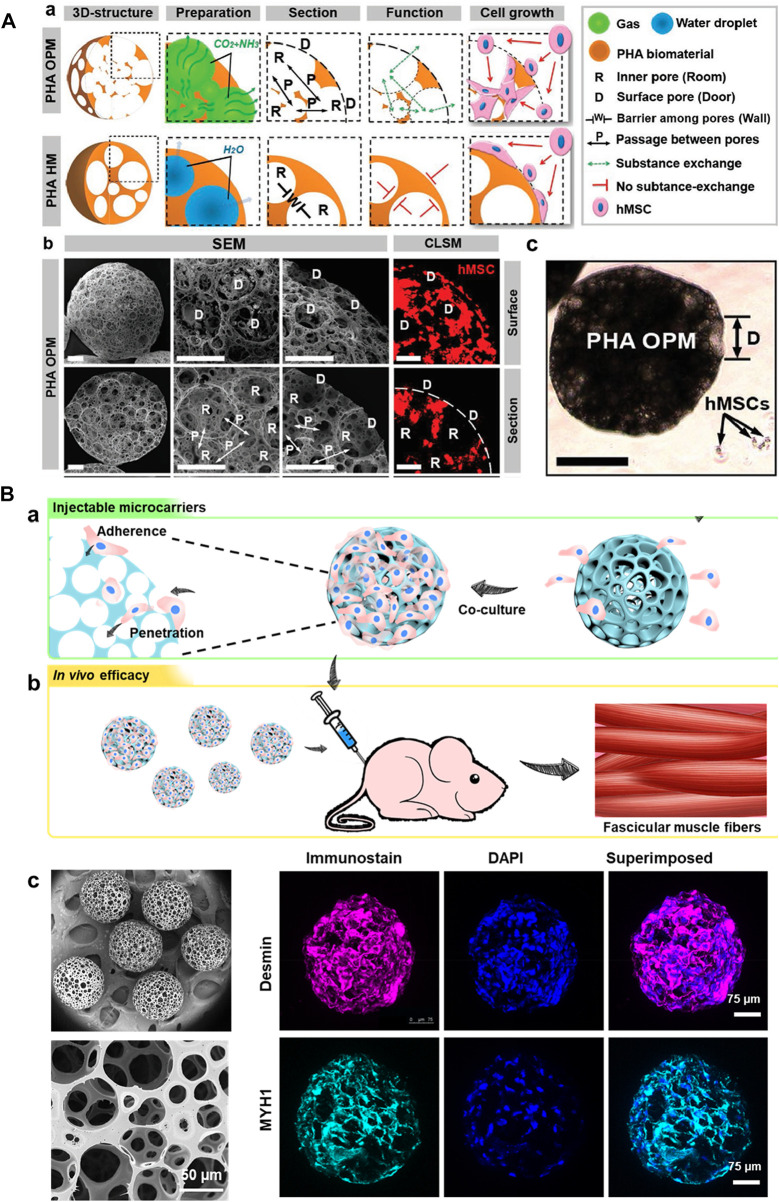
**(A)** Structures of PHA highly open PMs (PHA OPMs) and traditional PHA hollow microspheres (PHA HMs), respectively. a) Illustrations of PHA OPM and HM, including 3D structures, preparation, section, functions, and cell growth. b) SEM images of surfaces and sections of PHA OPM and confocal laser scanning microscopy (CLSM) images of hMSCs adhered to PHA HOPM. The bars are 50 μm. The actin of hMSC is stained in red. D: surface pore (door); R: inner pore (room); P: passage; and W: barrier among pores (wall). c) Sizes of hMSCs digested with trypsin versus a PHA HOPM. The bar is 150 μm. D: surface pore (door). Reproduced with permission from Ref. (Wei et al., 2018). Copyright 2018, John Wiley & Sons. **(B)** a) Schematic illustration showing the fabrication of modular cell-laden HOPMs by populating the C2C12 cells on the microcarriers *in vitro*, and b) evaluating their performance *in vivo* after administering these cell-laden HOPMs in nude mice. c) SEM images showing the size distribution of PLGA HOPMs, and surface morphology of a microcarrier as well as immunohistological analysis of myoblasts in the PLGA HOPMs by staining them against desmin and MYH1 (counterstained by DAPI for nuclei) for 7 days. Reproduced with permission from Ref. (Kankala et al., 2019), Copyright 2019, John Wiley & Sons.

In this context, various hydrophilic polymers, including PLGA, PHA, and PCL, among others, have been employed to generate these solidified microarchitectures (Hafeman et al., 2008; Wei et al., 2018; Liao et al., 2022). These solidified microparticles offer advantages of compatibility, excellent textural properties, and stability (thermal, colloidal, and suspension), which are of particular interest for minimally-invasive cell delivery towards TE applications (Hafeman et al., 2008; Jiang et al., 2016). The highly porous structures would substantially offer predominant encapsulation and proliferation abilities, leading to the subsequent convenience for their delivery in the injectable location (Wei et al., 2018). In a case, PHA-based open PMs (OPMs) with an average diameter of 300–360 μm were generated to avoid open surgery (Figure 2A) (Wei et al., 2018). These minimally-invasive scaffolding systems harbored with the proliferating stem cells (human mesenchymal stem cells, hMSCs) showed cell adhesion (93.4%) and proliferation efficiencies to repair the tissue defects. Compared with the PLA-based and hollow microcarriers, these PHA-based OPMs presented improved tissue restoration ability towards osteogenic regeneration. These PHA-based OPMs substantially presented the adhesion and encapsulation of skeletal myoblasts, indicating exceptional proliferation efficacy *in vitro* and regeneration capacity *in vivo*. In another instance, we generated PLGA-based microcarriers using the microfluidic approach in a two-step process for skeletal muscle cell delivery (Figure 2B) (Kankala et al., 2019). The resultant gelatin-assisted highly porous microcarriers based on PLGA enabled subsequent adhesion and infiltration of myoblasts in a more significant number. Then, immunohistochemical staining was performed using the myogenesis-specific biomarkers (myosin heavy chain (MYH) 1 and desmin). Finally, the subsequent delivery of myoblasts from the PLGA carriers resulted in tissue regeneration ability *in vivo*.

Although these solid microcarriers with porous connectivity in their interiors facilitate enough room for cell adhesion, infiltration, and proliferation, cells require certain additional supplements of biophysical and biochemical cues, such as growth factors and specific adhesion molecules (integrins, cadherins, and selectins)-ECM proteins (for example, fibronectin), to mimic the natural ECM-like microenvironment (Kim et al., 2015). In addition, strict utilization of hydrophilic polymers would substantially enable improved biocompatibility for cell adhesion and proliferation abilities for tissue repair and drug screening applications. For example, Li et al. (2014) reported the generation of alginate microspheres using the microemulsion and freeze-drying approaches. These microcarriers with anarchic microporous cavities on their surfaces resulted in the encapsulation of human hepatocellular carcinoma cells.

### 3.2 Micro-sized hydrogels

Due to advantageous hydrophilicity and ECM-mimicking features, micro-sized hydrogels have garnered interest from researchers in the delivery and recruitment of cells to promote TE (Bidarra et al., 2014; Oyama et al., 2014). Although similar to microparticles in terms of morphology, these water-rich gels with a 3D hydrophilic network, biocompatibility, and reactive chemistries make them appropriate for TE applications. These micro-sized hydrogels with polymeric building blocks are preferred over the solidified microparticles due to their tailorable physicochemical properties and similar ECM-like architecture (Bae et al., 2006). These building blocks are often engineered by crosslinking using various chemical reactions or physical interactions, forming the hydrogel in the presence of cells and proteins either during the fabrication or *in situ*. In addition to incorporating biological functionalities for modulating hydrogel properties, the ease of tailoring ability enables their applicability in the delivery of cells (Lavanya et al., 2020). Notably, the dimensions of hydrogels play a crucial role in the encapsulation of cells in their liquid environment, which, however, is often dependent on the engineering strategy and application requirements. Typically, the ideal size of the micro-sized gels should be around 200 μm in diameter with a volume of 1.0 nL to rapidly deliver gases and nutrients and excreting metabolic waste (Sheikhi et al., 2019).

Many polymers have been applied as building blocks to fabricate these polymeric injectable hydrogels. The compatibility and reaction chemistry attributes are crucial in selecting an appropriate polymer source as a building block. In addition, other specific criteria include crosslinking and subsequent degradability in the physiological fluids, as well as biochemical properties to facilitate cell growth. In this context, hydrophilic polymers are often preferred from natural and synthetic sources. The former type is derived from tissues or other natural origins, and the latter type is referred to as synthetic components attained through organic reactions (Velasco et al., 2012). The natural polymers for synthetic hydrogels include chitosan, hyaluronic acid (HA), keratin, heparin, fibrin, collagen, chondroitin sulfate, and alginate (Bidarra et al., 2014; Lavanya et al., 2020). These natural building blocks offer to replicate the native environment, including the mechanical and biochemical cues. Considering these aspects, the natural microgels offer advantages under mild conditions for minimally-invasive cell delivery towards RM (Wang et al., 2018). In a case, Kim and coworkers generated an alginate hydrogel system with a degradability attribute to encapsulate human adipose stem cells (hASCs) for engineering adipose tissues. Notably, the alginate was oxidized to improve the degradability and modified with the integrin-binding peptide (G4RGDASSKY) sequence to promote cell adhesion towards attaining cell-ECM interactions. These injectable hydrogels provided a suitable environment for cell growth and delivery for preconditioned cryopreserved hASCs to engineer adipose tissue. In another case, young and colleagues fabricated HA-based hydrogels for controlled survival, delivery, and differentiation of mouse retinal progenitor cells (Liu et al., 2013). The designed hydrogels were viscous, resulting in the ideal properties for transplanting and promoting the self-renewal and differentiation of retinal progenitor cells for retinal repair.

**FIGURE 3:**
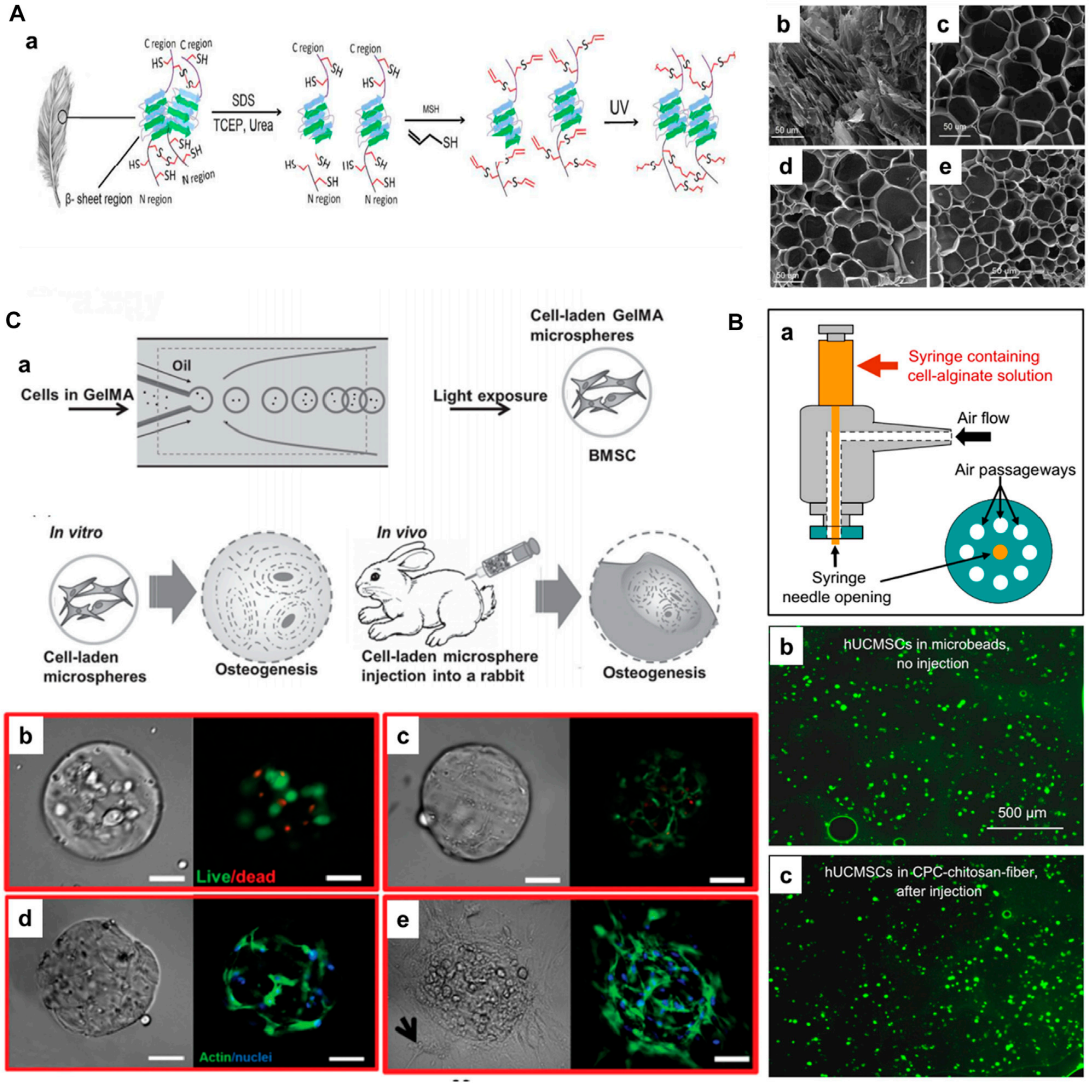
**(A)** a) Schematic illustrating the extraction of keratin and subsequent processing steps for preparing photocrosslinkable keratin hydrogels for stem cell encapsulation. SEM images of freeze-dried KeratATE precursor solution before ultraviolet (UV) crosslinking (b, 25 wt%) and after crosslinking with KeratATE concentrations of 15 (c), 20 (d), and 25 (e) wt% (scale bar in b–e is 50 μm). Reproduced with permission from Ref. (Barati et al., 2017), Copyright 2017, American chemical Society. **(B)** Schematic of the human umbilical cord MSCs (hUCMSCs)-encapsulating microbead synthesizer. hUCMSCs viability without injection or after injection. (b) hUCMSCs in microbeads (without CPC, without injection). (c) hUCMSCs in microbeads after mixing with CPC-chitosan-fiber paste and after injection. Reproduced with permission from Ref. (Zhao et al., 2010), Copyright 2010, Elsevier. **(C)** a) Schematic diagram of fabrication of BMSCs-laden gelatin methacryloyl (GelMA) microspheres and its application for osteogenesis and regeneration of injured bones. Photocrosslinking-microfluidic fabrication of GelMA microspheres and their encapsulated BMSCs differentiation and regeneration of bone *in vitro* and *in vivo*. Viability, spreading, and proliferation of BMSCs encapsulated in GelMA microspheres. b, c) Viability of BMSCs encapsulated in GelMA after b) 1 and c) 7 d of culture. Live (green) cells are labeled with calcein AM, and dead (red) cells are labeled with ethidium homodimer. d, e) Phalloidin/DAPI images of BMSCs cultured in GelMA after d) 2 and e) 4 weeks. Phalloidin stains cell filament green, and DAPI stains cell nuclei blue. Scale bar = 100 μm. Reproduced with permission from Ref. (Zhao et al., 2016), Copyright 2016, John Wiley & Sons.

Some natural hydrogels are functionalized to improve their cell-attachment motif (for example, keratin) and regulate the degradation (Du et al., 2015). In a case, Jabbari and colleagues demonstrated the generation of photocrosslinkable feather barbs keratin-based biopolymer for stem cell delivery towards tissue regeneration (Figure 3A-a) (Barati et al., 2017). Initially, the disulfide bonds were reduced in the extracted keratin from feather barbs. Then, the free thiols were converted to dehydrooalanine by oxidation and s-allyl cysteine in the presence of allyl mercaptan, resulting in the keratin allyl thioether (KeratATE) biopolymer. These biopolymeric honeycomb-like porous microgels with improved mechanical properties in the range of 1–8 kPa depending on the KeratATE concentration, resulted in the encapsulation and delivery of hMSCs, which turned out to be elongated spindle-shaped morphology after seeding into scaffolds (Figure 3A-b–e). These hydrogels showed improved proliferation of hMSCs, further supporting their differentiation to osteogenic and chondrogenic lineages. In another instance, Burdick and coworkers modified HA with aldehyde and hydrazine functional groups, enabling the modulation of myofibroblasts (Purcell et al., 2014).

To this end, the synthetic polymers for fabricating injectable hydrogels include poly(ethylene glycol), poly(vinyl alcohol) (PVA), poly(N-isopropyl acrylamide) (PNIPAAm), PEG, and PCL, among others, due to their low batch-to-batch variation, commercial availability, and ease of chemical modification, leading to controllable mechanical properties. Several synthetic polymers suffer from a major disadvantage of lack of inert biochemical cues. Among various synthetic polymers, PEG is one of the widely applied synthetic building blocks. PEG offers the major advantage of being relatively inert for introducing specific bioactive groups, for instance, modulating interactions with the cells through conjugating with acrylates or maleimides. In an instance, Phelps and coworkers generated PEG-maleimide matrices through maleimide cross-linking chemistry and peptides functionalized with thiols (Phelps et al., 2012). These hydrogels improved the viability of progenitor cells and promoted their spreading. Nevertheless, synthetic and natural polymers have been combined to improve the inherent biochemical cues toward improved cell interactions (Headen et al., 2014; Wieduwild et al., 2015; Wang et al., 2020).

In addition to chemical crosslinking, as evidenced in the aforementioned studies, physical crosslinking-based injectable hydrogels have been generated using various ionic, hydrogen bonding, and hydrophobic interactions, among others (Wang and Heilshorn, 2015). The most commonly used physical crosslinking-based injectable hydrogels are generated using ion- and/or temperature-induced gelation (Agarwal et al., 2013). Notably, synthetic polymers containing carboxylic acids, alcohols, and other side chains are often preferred (Du et al., 2015). In a case, Xu and colleagues generated alginate-based injectable hydrogels for encapsulating the human umbilical cord MSCs (hUCMSCs) for bone TE (Figure 3B) (Zhao et al., 2010). In another case, Sa-Lima and coworkers generated thermoresponsive PNIPAAm-g-methylcellulose (PNIPAAm-g-MC) as a 3D support for articular cartilage regeneration. Wang et al. (2017) demonstrated the covalently adjustable hybrid hydrogels based on the elastin-like protein–HA (ELP–HA) derivatives with secondary thermoresponsive crosslinking for minimally invasive delivery of stem cells. The combination of aldehyde-modified HA and hydrazine-modified ELP promoted improved mechanical properties (tuning the stiffness of the network in the range of 50–5,000 Pa) and substantially encapsulated MSCs for their delivery. Similarly, photo-crosslinking has garnered interest in TE and RM applications. The classic example of photocrosslinking of hydrogels includes the modification of gelatin with methacryloyl substituents, referred to as gelatin methacryloyl (GelMA), in the presence of light and photoinitiator. Gelatin, with abundant hydration properties and compatibility due to arginine–glycine–aspartic acid (RGD) residues, facilitate natural interactions with the cells and tissues. To improve the mechanical strength and gelation at physiological temperatures, the derivatization of gelatin to GelMA due to the photo-crosslinking property forms covalent links in the presence of a photoinitiator (Irgacure-2959). Notably, the degree of methacrylation can be regulated by controlling the amount of methacrylic anhydride. Owing to its considerable compatibility and controllable physicochemical properties, GelMA can be applied in TE applications, including cell delivery. Weitz and colleagues fabricated injectable photocrosslinkable microspheres based on GelMA for encapsulating bone marrow-derived MSCs (BMMSCs) towards osteogenic tissue constructs (Figure 3C) (Zhao et al., 2016). Notably, the GelMA could sustain stem cell viability, migration to the interiors, and substantial proliferation *in vitro* and *in vivo*. These minimally-invasive photocrosslinkable GelMA microspheres increased mineralization and facilitated bone regeneration. In another case, Hölzl et al. (2022) demonstrated the photo-crosslinked GelMA as a candidate for the delivery of chondrocytes. Similarly, Bae et al. (2006) generated HA-based hydrogel beads using the facile photo-polymerization of its methacrylated derivatives and N-vinylpyrrolidone using the alginate as a temporal mold. After optimization of conditions, such as methacrylated conditions and irradiation time, these beads were encapsulated with cells, resulting in a highly suitable environment for the viability and proliferation of bovine articular chondrocytes, suitable for minimally-invasive cell delivery applications.

In addition to polymeric hydrogels, several composite hydrogels with inorganic constructs have been reported to potentiate their minimally-invasive cell delivery applications toward tissue repair. In a case, magnetic microgels based on gelatin and PEG diacrylate (PEGDA) were prepared by cryogelation and micromolding approach for culturing HepaRG cells (Liu et al., 2014). These controllable MRI-traceable magnetic microtissues could be helpful in exploring tissue repair abilities and other critical issues in TE applications. In another instance, graphene oxide (GO)-encapsulated self-assembling peptide-based hydrogels for intervertebral disc repair (Ligorio et al., 2019). The use of GO fulfilled the applicability of nanofillers for reinforcing FEFKFEFK peptide hydrogel. Moreover, the strong interactions between the GO and hydrogel facilitated the mechanical properties, i.e., the storage modulus of >1,500 Pa compared to free hydrogel (<500 Pa), towards improving the cell viability as potential cell delivery scaffolds. Despite the success, these hydrogels suffer from several disadvantages, such as being fragile and lacking dynamic, as well as colloidal stabilities. While injecting cells, these attributes may lead to an inappropriate dosage of injectable cells at the administration site. Notably, these water-rich hydrogels enable convenience for fabricating biological substitutes (organoids and spheroids) and investigating cell-cell interactions. Further, the performance of delivery and subsequent tissue repair and regeneration abilities are dependent on various cellular and environmental factors.

### 3.3 Cells encapsulated with nanocomposites

**FIGURE 4:**
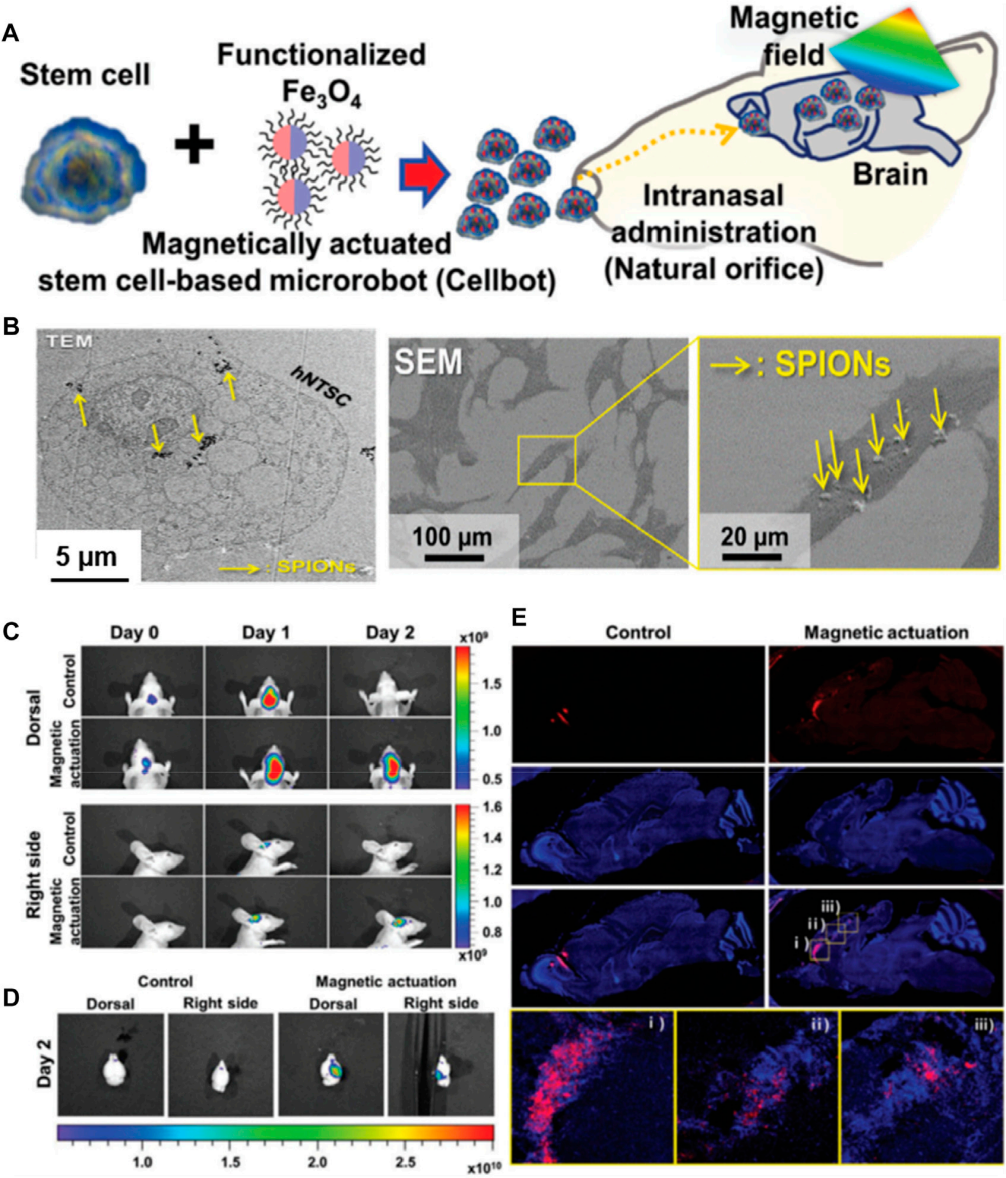
**(A)** Schematic of the intranasal administration and magnetic actuation of Cellbots. **(B)** TEM image visualizing cellular uptake of PLL-SPIONs in the cytoplasm and SEM images of the SPION-labeled hNTSCs (arrows indicate PLL-SPIONs). *In vivo* delivery of Cellbots into the target brain region *via* intranasal administration and magnetic guidance. **(C)** *In vivo* imaging of the control group (without magnetic field) and magnetic actuation group (with magnetic field) for 3 days. **(D)** Fluorescence images of the extracted mouse brain after 2 days. **(E)** Sequential migration and engraftment of the cell bots from the injection site (olfactory bulb) to the cerebral cortex. Reproduced with permission from Ref. (Jeon et al., 2021). Copyright 2021, John Wiley & Sons.

In addition to encapsulating cells in micron-sized particles and gels, guided delivery can be achieved by internalizing various nanocomposites into the cells. These small-sized sub-micron bots can be employed for guiding toward minimally-invasive cell delivery. Although these materials are in the sub-micron size range, it is worth noting that the internalized constructs would substantially facilitate the transportation of cells toward the target site, similar to the polymeric microcarriers. In a case, cell bots were generated by Jeon and colleagues by internalizing the superparamagnetic iron oxide nanoparticles (SPIONs) into human nasal turbinate stem cells (hNTSCs) (Figure 4) (Jeon et al., 2021). These cell bots were constructed by culturing the hNTSCs with the poly-_L_-lysine (PLL)-coated SPIONs with highly positive-charged amino acid chains for minimally-invasive targeted delivery of stem cells to the brain tissues. The intranasal administration of cell bots was targeted by magnetic actuation using the external magnetic field, which could be efficient for treating central nervous system (CNS)-based diseases in terms of therapeutic delivery.

## 4 Microfabrication strategies

Broadly speaking, several conventional biofabrication approaches for recreating such building blocks predominantly include top-down and bottom-up approaches (Santana et al., 2020). These approaches aim to recapitulate the complex 3D architectures that mimic the tissue-specific environment and their bio-functionalities. In the top-down processing-based approaches, cells are expected to be populated in support of the fabricated porous scaffolds towards recapitulating the native ECM, including the biochemical and physicochemical cues (Langer and Vacanti, 1993). To accomplish these tasks, several scaffolding systems have been fabricated to mimic the tissue microarchitecture, anatomical, and physiological features along with the spatiotemporal rearrangements of ECM. Numerous technologies, including lithography (photo-/soft-), two-photon polymerization, and 3D printing, among others, are often practiced for fabricating such 3D architectures (Choi et al., 2012). Furthermore, mechanical stimulants or enhancers, such as growth factors, regulate tissue biology involving mechanical and biomolecular signaling attributes. Despite the progress, these approaches often suffer from various limitations, such as challenges in cell immobilization in the scaffolds, resulting in low yield and irregularities in their spatial distribution, and failing to mimic unit-repetitive modular designs in derived ECM similar to native tissues. To this end, diverse types of bottom-up approaches have emerged as promising alternatives to top-down approaches for developmental biology toward fabricating bioinspired components (Kankala et al., 2018a). This bottom-up biofabrication offers a unique design of building blocks of highly flexible arbitrary-sized constructs in the range of atomic scale to supramolecular large-sized assemblies (Choi et al., 2012). The cellular-rich building blocks of polymeric carriers can be built through self- or guided cell assembly feasibly using the controlled distribution of different types of cells. Despite the availability of various approaches, several challenges exist to fabricating microcarriers with uniform size distribution and controllable, as well as reproducible morphologies. This section presents different microfabrication strategies for generating microcarriers, highlighting the pros and cons of harboring cells and their subsequent delivery.

### 4.1 Microfluidic technology

Droplet microfluidics refers to an approach of perceiving behavior and precise manipulation of fluids (10^−9^–10^−18^ L) on the microscale at which surface forces take control of the volumetric forces towards generating the microminiaturized devices (Bhatia and Ingber, 2014). This multidisciplinary field is practically utilized in systems that process low volumes of fluids to achieve automation, multiplexing, and high-throughput screening, by integrating various areas of physics, engineering, chemistry, and nanotechnological concepts. Although various traditional microfabrication technologies are available, droplet microfluidics has garnered captivating interest for generating microparticles due to the tunability of the composition, controllable geometrical topographies, and high-throughput generation (Bhatia and Ingber, 2014; Jiang et al., 2016). Typically, the first-applied microfluidic devices in the 1980s were designed using silicon and glass. Typically, the emulsion droplets in the microfluidics process, one at a time, are initially generated using the device set-up through the careful balance between various forces, including inertial forces, interfacial tension, and viscous (Ofner et al., 2017). Besides, other external forces (centrifugal, magnetic, and electric) are applied to generate droplets. The droplet generation plays a vital role in the eventual quality of the microcarriers, as the precise manipulation of fluid miscibility would influence the outcome. In addition, other factors play substantial roles in the processing and quality of microcarriers, such as channel geometries and flow conditions. In this context, various channel geometries (co-flow, T-junction, and flow-focusing), along with the parallelization of multiple channels, would result in the generation of uniform-sized droplets with high-throughput efficiency (Garstecki et al., 2006; Ofner et al., 2017). The well-defined geometry of the device set-up and compatibility are crucial in generating emulsion droplets. The glass capillary-based microfluidic setup is predominantly used due to high chemical resistance and ideal co-axial fabrication of droplets. Despite the success, reproducibility and scale-up issues yet remain to be addressed (Benson et al., 2013). To a considerable extent, soft lithography-based polydimethylsiloxane (PDMS) and polytetrafluoroethylene (PTFE)-based microfluidic devices have emerged due to elasticity, compatibility, optical transparency, and non-inflammability (Xia and Whitesides, 1998; Bhattacharjee et al., 2016). Several reviews explored detailed mechanistic insights based on droplet generation involving dynamic forces and their effects on the outcome (Zhu and Wang, 2017).

**FIGURE 5:**
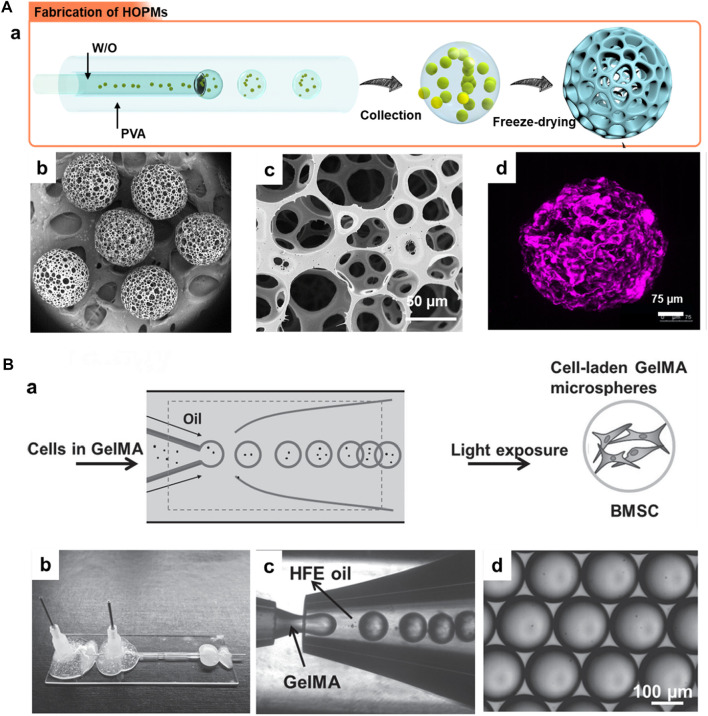
**(A)** a) Schematic illustration showing the generation of PLGA HOPMs by microfluidic technology towards the fabrication of modular cell-laden HOPMs by populating the C2C12 cells on the microcarriers. b) SEM images showing the size distribution of PLGA HOPMs, and c) surface morphology of a microcarrier. d) immunohistological analysis of myoblasts in the PLGA HOPMs by staining them against desmin for 7 days. Reproduced with permission from Ref. (Kankala et al., 2019), Copyright 2019, John Wiley & Sons. **(B)** a) Schematic diagram illustrating the photocrosslinking-microfluidic fabrication of GelMA microspheres encapsulated with BMSCs. Aqueous droplets containing GelMA gel precursors are produced from a microfluidic flow-focusing device and photopolymerized to form GelMA microspheres. b) A Photograph of the microfluidic device, c) a microscope image of the device generating GelMA droplets, and d) monodisperse GelMA droplets in HFE oil. Reproduced with permission from Ref. (Zhao et al., 2016), Copyright 2016, John Wiley & Sons.

Using the microfluidics technology, the cells-encapsulated microcarriers for cell delivery applications can be fabricated in two ways, direct and indirect approaches. In the direct encapsulation strategy, the cells are incorporated in the emulsion droplets and further subjected to the solidification of polymers-encapsulated cells by cross-linking (Yanagawa et al., 2016; Siltanen et al., 2017). Numerous advantages include the formation of uniform-sized particles, convenience in regulating the physicochemical properties, and control over the cell encapsulation efficiency (Siltanen et al., 2017). Despite the success in generating cells-encapsulated microcarriers, maintaining the extensive surface area and porosity is often required, facilitating the convenience of exchanging nutrients and gases for substantial growth and delivery of cells (Yanagawa et al., 2016; Li et al., 2018). In addition to the one-step approach, a two-step indirect approach has been employed to fabricate cell-encapsulated microcarriers. The biocompatible polymeric carriers (both porous and non-porous) can be initially generated using the microfluidic technology, which are then cultured with the desired cells of interest for a specified time period, for instance, 14–28 days (Leong et al., 2003). Despite the multi-step processing and time-consuming, this approach presents the additional advantage of the ease of mounting the cells in the carriers. These carriers offer a conducive microenvironment for the growth and proliferation of cells, depending on the porosity, biocompatibility, and hydrophilicity of the carriers (Loh and Choong, 2013; Chen et al., 2015). For instance, our group has generated PLGA-based microcarriers using the microfluidic approach in a two-step process for skeletal muscle cell delivery (Figure 5A-a) (Kankala et al., 2019). Firstly, the microfluidics resulted in highly porous architectures (average diameter of >300 µm and porosity range of 10–80 µm) with the support of gelatin as a porogen (Figure 5A-b–d). In addition to polymeric solid microcarriers, the microfluidic approach can generate liquid-rich micro-sized injectable hydrogels. Although various materials are available, including natural (alginate) and synthetic (PEGDA) polymers, these materials lack cell-responsive motifs, such as anchorage proteins. Considering these issues, in a case, Zhao and colleagues generated hydrogel microspheres using the microfluidic approach to encapsulate BMSCs and their subsequent delivery to promote osteogenesis *in vitro* and *in vivo* (Figure 5B). Initially, the droplets of GelMA, photocrosslinkable gelatin, and photoinitiator for photopolymerization were introduced into the microfluidic device. Further, the polymerization of the droplets resulted in the fabrication of monodisperse GelMA microspheres with an average size of over 165 μm. As specified in the advantages of injectable microgels in Section 3, the gentle gelling condition could substantially minimize the damage to the encapsulated proteins and cells in the microgels. These microgels substantially promoted the viability of BMSCs and spread inside the microgels.

### 4.2 Emulsification

Although the microfluidics technique is predominantly based on the emulsification of droplets towards the fabrication of microcarriers (ofner et al., 2017), the polymeric microcarriers can be generated using the facile emulsification process without microfluidics. In general, the fabrication process is based on the initial emulsification (W/O or O/W) of the droplets containing polymers and porogens followed by the mineralization, using crosslinking with ions, freeze-drying, and thermal-induced phase separation (Li et al., 2015; Zhang et al., 2017). Similar to microfluidics, several polymers can be used to fabricate cell-laden microcarriers, including cellulose, collagen, alginate, chitosan, and PLGA (Zhang et al., 2017; Huang et al., 2018). In an instance, Zhang et al. (2017) generated cellulose-based aerogel microspheres using the single emulsification approach followed by freeze-drying. These cellulose microspheres provided abundant cell surface area for the adhesion and proliferation abilities of the mouse fibroblasts (NIH/3T3). Similarly, the emulsion (O/W)-based porogen leaching-phase separation processes were implemented to fabricate the PLGA-based microcarriers, which showed tremendous cell encapsulation efficiency (Chung and Park, 2009). Fernandes Patrício et al. (2019) generated collagen-based superparamagnetic microspheres, which were further mineralized using (Fe^2+^/Fe^3+^)-doped hydroxyapatite (HAp) and emulsified using citrate species. The resultant carriers displayed a suitable microenvironment for the growth of mouse pre-osteoblast cell line (MC3T3-E1), showing cytocompatibility and subsequent osteogenesis. In another instance, chitosan microspheres with high porosity were fabricated for 3D culturing of cells using the micro-emulsification approach followed by the thermally-induced phase separation (Huang et al., 2018). In addition to the single-emulsion-based method, several attempts based on the double-emulsification approach have been made to fabricate cell-laden microcarriers. For instance, Nilsson et al. (1986) applied the double emulsification method followed by freeze-drying to encapsulate various animal cells (Baby Hamster Kidney cells, BHK, and African green monkey cells, VERO). Similar to generating various cell-laden microcarriers for cell delivery and tissue repair applications, several efforts have been dedicated to generating cells-encapsulated tumor models (pancreatic ductal adenocarcinoma (PDAC) using the emulsification-assisted photo-crosslinking methods to reiterate the TME for drug screening applications (Brancato et al., 2017; Pradhan et al., 2017).

### 4.3 Self-assembly approach

The self-assembly process, a bottom-up approach, is often referred to as the organization of various disordered species to supramolecular highly organized architectures (Miller et al., 2021). This approach often depends on diverse interactions between the precursors, such as hydrogen bonding, capillary, and van der Waals, resulting in fabricating architectures with varied dimensions (Salem et al., 2003; Oyama et al., 2014). Although these notified interactions are independent of the charge of the precursors, the overall surface charge of the components may facilitate their convenient assembly. For instance, contrarily charged species enable the ionic interactions substantially augment the assembly process (Ligorio et al., 2019). As cells are negatively-charged, the positively-charged material surfaces, for instance, chitosan and PLL, conveniently facilitate their assembly as composite scaffolds (Salem et al., 2003; Huang et al., 2018). In a case, Jeon et al. (2021) generated targeted cell bots in which hNTSCs were internalized with SPIONs for minimally-invasive delivery of stem cells to brain tissues. These cell bots were fabricated by culturing the cells with the PLL-coated SPIONs with highly positive charged amino acid chains. These composites with insignificant toxicity were substantially internalized into the hNTSCs, and employed for minimally-invasive targeting of stem cells to brain tissues. The intranasal administration of cell bots was targeted by magnetic actuation using the external magnetic field, which could be efficient for treating CNS-based diseases in terms of therapeutic delivery. In another instance, a simple solvent-exchange-assisted lyophilization was applied to generate nanofibrous hollow microspheres through the self-assembly of star-shaped polymeric constructs as minimally-invasive carriers of cells for knee repair (Liu et al., 2011). These self-assembled nanofibrous microcarriers showed improved adhesion and proliferation of chondrocytes *in vitro* and *in vivo*, presenting osteochondral repair. Although this process is often used to fabricate microarchitectures, obtaining uniform-sized architectures is challenging, requiring control over all the parameters, and the need for high temperatures to dissolve polymers is inevitable.

### 4.4 Isothermal spherulitic crystallization

Recently, a unique microfabrication strategy, named isothermal spherulitic crystallization, has been proposed to generate microcarriers. These generated microcarriers exhibit several advantages, such as convenience for operation, simple set-up, scale-up, and adaptability (Kuterbekov et al., 2018). In addition, the major advantage of this approach is the non-utilization of organic solvents, which would be convenient for the adhesion and growth of cells due to the biocompatibility of resultant microcarriers. In a case, PLA-based porous architectures were synthesized using the organic solvent-free spherulitic crystallization approach, resulting in the regulated morphological attribute in terms of particle size and pore diameter (Kuterbekov et al., 2018). The resultant microcarriers were highly biocompatible to culture hASCs, exhibiting potential in adhesion and growth, and differentiation abilities of hASCs.

### 4.5 Graft polymerization

Graft polymerization refers to synthesizing polymerized constructs by imbedding monomers with covalent linkages as side chains onto the main polymer, resulting in the altered polymer composite. This approach has been applied to impart various chemical functionalities to the polymeric chains, for instance, hydrophilicity to hydrophobic polymers and vice versa (Le et al., 2019). Often, this method can be applied to alter the surface chemistry and morphological attributes of the polymeric constructs. Plasma-induced grafting, one of the grafting-based approaches, has attracted captivating attention to alter surface morphology (Liu et al., 2022). This approach acts by polymerizing the surface of a plasma-activated polymer, resulting in brush-like polymeric layered surfaces. The highly active grafted surfaces can extend several nanometers of depth, facilitating the immobilization of cells and biomolecules, for instance, proteins. These active surfaces can enable the interactions between the protein chains on the cellular surfaces and polymeric functional groups on the layers. In an instance, acrylic acid was graft-polymerized onto plasma-treated poly(ethylene terephthalate) (PET) films to facilitate the surfaces feasible for collagen immobilization and further seeding smooth muscle cells (Gupta et al., 2002). The collagen immobilization efficiency increased with the grafting density of the films. Finally, the collagen-immobilized grafts were tested for seeding the smooth muscle cells, resulting in the improved growth of smooth muscle cells.

### 4.6 Miscellaneous

Various other techniques have recently garnered interest in fabricating various carriers for cell delivery and subsequent TE applications. Although some of the techniques are not directly related to cell delivery, the discussions related to such techniques are worth discussing their potential towards injectable constructs for TE applications, for instance, electrospraying (Nilforoushzadeh et al., 2020). In this section, we present discussions relevant to some of these techniques, such as electrospray, molding, and acid-dissolved/alkali-solidified self-sphering shaping methods.

**FIGURE 6:**
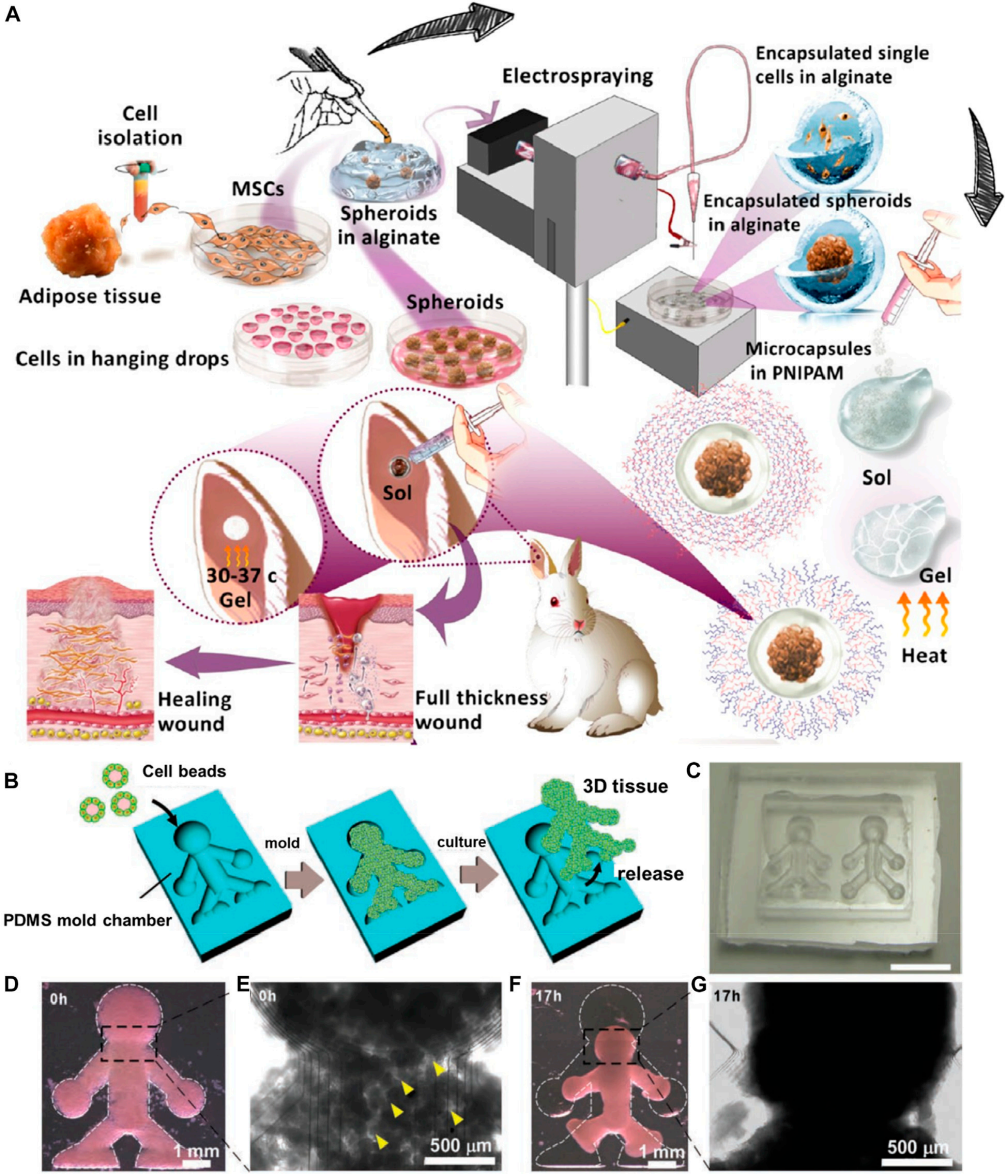
**(A)** Schematic illustration of the electrospray setup used for microencapsulation. Reproduced with permission from Ref. (Nilforoushzadeh et al., 2020). Copyright 2020, American Chemical Society. **(B)** The method used to produce the 3D tissue architectures using monodisperse cell beads. **(C)** A microscopy image of the doll-shaped PDMS mold chamber reveals 3D tissue formation. **(D,E)** Microscopy images of NIH/3T3 cell beads immediately after stacking. Cavities (indicated with yellow arrows) among the cell beads are observed at this time point (0 h). **(F,G)** Microscopy images of NIH/3T3 cell beads, 17 h after stacking. Reproduced with permission from Ref. (Matsunaga et al., 2011). Copyright 2011, John Wiley & sons.

Electrospraying, referred to as electrohydrodynamic atomization, is a voltage-driven approach capable of producing micro- and nano-sized particles. The liquid/polymeric solution is sprayed through the nozzle in the presence of high electrical forces. This cost-effective approach offers several significant advantages of altering the processing parameters to regulate the resultant particles, such as the distance between the collector and nozzle and the applied voltage (Wang et al., 2018; Clohessy et al., 2020; Miller et al., 2021). In a case, Mozari et al. initially generated 3D spheroids of MSCs, which were then encapsulated using the electrospray technique in the micro-scale alginate beads and subsequently into the injectable thermosensitive PNIPAAm-based hydrogel matrices (Figure 6A) (Nilforoushzadeh et al., 2020). These gels could dissociate at the skin temperature, delivering cells, sealing the wound cavities, and protecting the alginate beads from the harsh wound environment. Several investigations *in vitro* and *in vivo* were performed to determine the secretion of various biological mediators, such as α-smooth muscle actin (α-SMA) and transforming growth factor β1 (TGF-β1), toward effective cell-based wound therapies. In another instance, Tian and coworkers demonstrated the generation of injectable gelatin methacryloyl-alginate core-shell microcapsules using the coaxial electrostatic microdroplet approach. These carriers efficiently delivered co-encapsulated human dental pulp stem cells (hDPSCs) and human umbilical vein endothelial cells (HUVECs) for endodontic regeneration (Liang et al., 2022). These constructs showed promotion of osteo/odontogenic differentiation along with vascularization in the microcapsules, resulting in the deposited ECM. Similarly, Peng and colleagues utilized the electrospray technique to prepare alginate-gelatin microspheres embedded with adipose-derived stem cells for cartilage tissue regeneration (Liao et al., 2022).

Indeed, the electrospray approach has garnered interest from researchers due to the ease of control over the mass production of micro-sized particles. Moreover, the generation of solid microspheres may suffer from poor cell encapsulation efficiency. However, nanofibrous and highly porous polymeric constructs are challenging to obtain using the electrospray approach alone. To address this limitation, Xie and colleagues used a combination of electrospinning and electrospraying approaches to generate injectable nanofibrous microspheres (Boda et al., 2018; John et al., 2020). In a case, electrospun aligned PCL-gelatin and PLGA-gelatin fibrous segments were electrosprayed (voltage = 8–10 kV, flow rate = 2.0 mL/h, and distance of 10 cm) into injectable nanofibrous microspheres for minimally-invasive cell therapy (Boda et al., 2018). These microspheres exhibited improved stem cell proliferation and differentiation efficiencies compared to solidified microparticles.

In addition to the aforementioned significant approaches, several other approaches have been employed to explore the generation of injectable modular units with the potential of cell delivery, such as molding (Bae et al., 2006; Hafeman et al., 2008; Lim et al., 2009). Molding can generate 3D architectures at arbitrary sizes based on mold dimensions. In a case, PCL microspheres with large pores as minimally-invasive cell delivery carriers were generated using the liquid mold room temperature ionic liquid (RTIL) and porogen camphene for microsphere development (Kim et al., 2016). The microspheres were modified with nerve growth factors on their surface along with gelatin to improve the attachment and delivery of neural progenitor cells (PC-12). In another instance, moldable bone substitutes based on sodium alginate (SA)/*β*-Tricalcium phosphate (β-TCP) microspheres cross-linked with an aqueous calcium chloride solution were generated for improved osteoconductivity toward curing bone defects (Ho et al., 2020). In addition, a micromolding approach based on the on-chip technology was applied to generate magnetic microcryogels-assisting microtissue formation with improved robustness and controllability, which could be applied for TE and drug screening applications (Liu et al., 2014). These carriers after delivering cells would facilitate their bottom-up assembly into well-organized structures (Luetchford et al., 2020).

Despite the generation of carriers based on size and shape interest, it often results in large-sized constructs. However, it is worth discussing that the resultant structures often provide enough space for fabricating complex architecture for designing the ECM-mimicking environment for cell growth. In these circumstances, the pre-designed microcarriers using other approaches have been adapted to molding approaches to generate complex structures (Wu et al., 2018). In an instance, Matsunaga et al. (2011) initially generated cells-coated collagen microbeads using microfluidics, which were further deposited in the silicone chamber as a mold (Figures 6B–G). Although the mold suggested the boundary for the growth of cells, the collagen beads supported the cell adherence, growth, and proliferation, resulting in the 3D microtissues.

Recently, an acid-dissolved/alkali-solidified self-shaping approach was proposed to generate 3D microcarriers (Zhang et al., 2018). This self-shaping strategy presents advantages, such as ease of operation, mono-disperse end products, and cost-effectiveness. In an instance, chitosan-based microcarriers were fabricated and reinforced with GO (Zhang et al., 2018). The resultant microcarriers displayed biocompatibility, and adhesion, as well as proliferation abilities to hUCMSCs for their long-term survival and differentiation capabilities. In another instance, PCL-based microscaffolds were prepared using the combinatorial approach. The isolated particle-melting method resulted in the non-porous beads and melt-molding particulate-leaching approach for obtaining the porous beads in the size range of 400–550 μm (Bidarra et al., 2014). These biocompatible beads displayed encapsulation efficacy of chondrocytes and their infiltration for cell delivery applications. Although most of these techniques have been utilized to generate 3D microcarriers with cell encapsulation ability, these techniques remained on the lab scale, require further parameter optimization and subsequent exploration on various cell types yet remain to be explored comprehensively.

## 5 Factors influencing cell delivery

According to a formulator anticipation, the designed carriers must offer ideal delivery attributes, specifically in terms of precise and controlled delivery of drugs and biomolecules (growth factors). Regarding cell delivery, the ideal carriers must possess specific abilities, such as high encapsulation of viable cells and their subsequent delivery abilities at the target site, promoting tissue regeneration. In this context, several factors predominantly influence the offered abilities by the carriers for cell delivery, such as type of polymer (source and functionalities), morphology (size, porosity, and shape), and injectability. This section presents a brief overview of these factors, highlighting their influence on the fabrication, encapsulation, and delivery of cells toward tissue repair.

### 5.1 Type of polymer

Owing to the successful encapsulation of cells in a viable form, the biocompatibility of the polymer substantially attributes to its selection process, depending on the nature and chemical composition of the raw materials, i.e., polymers. To a considerable extent, the selection of polymer mainly plays a vital role in fabricating microcarriers for cell delivery. Based on the source of origination, different kinds of polymers have been employed to fabricate these 3D microcarriers (non-porous and porous) for cell delivery applications, such as natural (gelatin, dextran, cellulose, chitin and its derivatives, and alginic acid) (Bidarra et al., 2014; Ho et al., 2020; Lavanya et al., 2020; Rahman et al., 2021; Liang et al., 2022; Liao et al., 2022), and synthetic (PLGA, PLA, silk fibroin, SF, polyurethane, polyacrylamide, and PHEMA) (Hong et al., 2005; Hafeman et al., 2008; Braccini et al., 2020). Considering the pros and cons, polymers from natural sources possess high biocompatibility due to biomolecules, recyclability, and mechanical properties (Rahman et al., 2021). These natural polymers significantly facilitate the conduciveness for forming an ECM-like microenvironment concerning the composition of polysaccharides and other biomolecules (Wang et al., 2021). Synthetic polymers offer mechanical attributes, reproducibility, tunable physicochemical features, and alterable morphological attributes of the eventual 3D microcarriers (John et al., 2020). Despite the advantageous features, some polymers would hinder the encapsulation and growth of cells in the interiors of cells due to depriving compatibility and non-favorable chemical composition, failing to form an ECM-like environment and severely affecting the adhesion and growth of the encapsulated cells. In comparison between natural and synthetic polymers, natural polymers offer improved biocompatibility, while synthetic polymers present improved mechanical properties. Considerably, the combination of synthetic and natural polymers at an appropriate proportion would substantially lead to the developing of excellent microcarriers with improved biocompatibility and appropriate mechanical properties. These features facilitate conducive encapsulation and proliferation abilities of cells in the interiors and on the surface of the designed microcarriers (Wang et al., 2011).

### 5.2 Morphology

In addition to the selection of raw materials, the predominant morphological attributes (for instance, size and shape) of resultant polymeric architectures play substantial roles in developing injectable microarchitectures and their subsequent TE applications (Li et al., 2018). The eventual particle/microgel size is one of the predominant factors of morphological features of microarchitectures, as the size quality plays a critical role in their injectability. In general, the acceptable size range of microarchitectures suitable for the delivery of cells is in the range of 100–500 μm. This optimal size and spherical-shaped containers range substantially facilitate the ample amount of cells in the interiors of the microarchitectures (Hong et al., 2005; Wei et al., 2018). In the case of hydrogels, the ideal size of the microgels is around 200 μm and a volume of 1 nL to achieve the rapid delivery of nutrients and water, as well as gases exchange in the interiors for the survival of the encapsulated cells (Vasile et al., 2020; Wang et al., 2021). In addition to improved encapsulation, these morphological attributes would avoid avoiding cell necrosis in their interiors.

### 5.3 Porosity

In addition to morphological attributes, textural properties, such as surface texture and porosity, play critical roles in encapsulating diverse cell types and their delivery. Initially, the rough surface texture provides feasibility in improving the adhesion efficiency of cells. In this context, the fabricated biocompatible solid microspheres with rough surfaces and tiny pores provided improved adhesion efficiency on their surfaces compared to the smooth surfaces (Matsushita, 2020). The plausible reason for improved adhesion by rough surfaces could be the non-slippery interactions between the cells and microcarriers. Although the rough surface enables improved adhesion, these solid microcarriers suffer from a significant limitation of low encapsulation yields due to less surface area (Choi et al., 2012). It is often required to provide extensive porosity with highly open and interconnected windows to enable improved encapsulation efficacy of different cell types. Considerably, the rough surfaces and highly open porosity facilitates improved adhesion and encapsulation efficacy, determining their delivery efficacy. Several biofabrication approaches have been employed to develop microarchitectures with high and controlled porosity with open and interconnecting windows to address these issues. Generally, the ideal porosity of the microparticles of an average diameter of 300 μm must be in the range of 10–100 μm (Choi et al., 2012). The plausible reason for these PMs with heterogeneous porosity is due to the high surface area. The abundant porosity of the carriers enables the exchange of gases and nutrients for the improved proliferation of cells in interiors (Kankala et al., 2019). In addition, the porous architectures substantially facilitate the cells encapsulating in the interiors and delivering to the tissue region of interest.

### 5.4 Surface charge

Indeed, several physicochemical characteristics of the raw materials determine various attributes of microcarriers; for instance, the chemical composition of the precursor defines the compatibility of the particles (Wang et al., 2021). Among various such characteristics, hydrophilicity and surface charge determine the adhesion and growth of cells in the carriers. In this vein, the selection of polymer, along with various surface-altering approaches (surface grafting, chemical modification, and plasma functionalization), have been applied to improve the affinity of the surface (Gupta et al., 2002). Despite the success in improving the surfaces for better adhesion of cells compared to unaltered surfaces, these approaches require multi-functionalization steps, which may alter the robustness and durability of the carriers (Wang et al., 2021). To this end, tissue-derived ECM has been developed to avoid these issues, for instance, decellularized adipose tissue, and micronized acellular dermal matrix. In addition, these support by offering biocompatibility and proliferation efficiency due to the natural ECM.

In addition to the factors mentioned above, several other factors play significant roles in influencing encapsulation and delivery efficiencies, for instance, injectability. In general, the delivery of the cells often depends on the target site, which is often designed based on the route of administration and injectability attributes. Preferably, intramuscular and intravenous injection routes are often used to deliver cells. Notably, the material characteristics and the cellularized secretions can generate and mimic the ECM-like environment (Hollister, 2005; Choi et al., 2010; Choi et al., 2012). However, various biochemical cues are required along with the desired microarchitectures as prerequisites for TE to control the microenvironment substantially.

## 6 Scope for preclinical/clinical applicability

Owing to their morphological attributes and textural properties, these polymer-based micron-sized carriers (solidified porous and non-porous carriers, as well as liquid-rich hydrogels) offer numerous advantages such as widespread cell encapsulation and carrying abilities, biodegradability, and biocompatibility, which are of unique interest for various biomedical applications. Compared to large-sized scaffolds (photo-cross-linkable hydrogels and biodegradable scaffolding systems) that require sophisticated fabrication steps, and highly invasive surgical procedures for implantation, these microcarriers facilitate room for the encapsulation of different cell types for tissue growth. Nevertheless, it should be noted that different carriers offer some unique attributes. For instance, solidified carriers with interconnecting windows facilitate enough room for the infiltration of cells and their subsequent metabolic activities, requiring additional elements or altered surfaces to provide an ECM-like environment. Contrarily, the liquid-rich hydrogels provide an abundant hydrophilic environment similar to a natural tissue-like microenvironment.

Before discussing the preclinical outcomes and scope for clinical translation, it is necessary to understand various attributes, such as biocompatibility and biodegradability. In most of the instances, several polymers with compatibility have been demonstrated, for instance, natural [chitosan (Huang et al., 2018), HA (Bae et al., 2006), and alginate (Chen et al., 2015)], and synthetic [PLGA (Kankala et al., 2019), PLA (Liu et al., 2011), and PCL (Kim et al., 2016)], as well as their mixture (Hong et al., 2005). In this framework, several cell lines from humans and mice have been encapsulated to demonstrate the potential of the microcarriers, such as osteoblasts (Chen et al., 2015), skeletal myoblasts (Kankala et al., 2019), chondrocytes (Hong et al., 2005; Liu et al., 2011), and MSCs (Liu et al., 2011; Barati et al., 2017; Luetchford et al., 2020; Liao et al., 2022), indicating their biocompatibility due to viability and proliferation abilities (Ho et al., 2020). Although these carriers showed compatibility with different cell lines, comprehensive toxicity evaluations must be systematically evaluated, including the genotoxicity and other toxicity evaluations. In some instances, the degradability attribute of the designed microcarriers, specifically solidified microcarriers, was demonstrated *in vitro*, requiring further investigations to explore the time of degradation and validations for degraded products. Further, the biocompatibility along with performance attributes have been evaluated *in vivo* in mice (Wei et al., 2018). In addition, these carriers with hydrophilicity and surface charge often result in the degradability *in vivo*, indicating no damage to the major organs (Kankala et al., 2019).

Although various cell lines have been used to explore the potential of such microcarriers as cell delivery vehicles, specific polymers for some specific cell lines have been used predominantly. In this framework, polymers from natural and synthetic origins have been applied. However, some polymers have been often applied, such as alginate, keratin, and PCL, in the solidified carriers and gelatin-based hydrogels as liquid-rich microgels (Wang et al., 2018). In some instances, the combination of natural and synthetic polymers has also been employed to generate hydrogels and solidified carriers, such as collagen-PLL (Hong et al., 2005), chitosan-PLGA (Wang et al., 2011), and gelatin-PCL (Boda et al., 2018). The predominant reasons behind the selection of polymers might be the hydrophilicity and compatibility attributes. To this end, several cell lines have been used to generate microcarriers for tissue repair. To a considerable extent, only a few kinds of cell lines have been abundantly studied, such as MSCs (Liu et al., 2011; Barati et al., 2017; Luetchford et al., 2020; Liao et al., 2022), osteoblasts (Chen et al., 2015), skeletal myoblasts (Kankala et al., 2019), and chondrocytes (Hong et al., 2005; Liu et al., 2011). In most instances, the MSCs (human/rat BMMSCs) have been intended to deliver them into the appropriate region of interest and explore their proliferation and differentiation efficiencies. Moreover, the specific reason for selecting such cell lines could be due to ease of growth and infiltration and the route of administration, i.e., minimally invasive. Eventually, the preference in selecting polymer and cell line remains arbitrary, depending on the specific attributes of applicability, cell morphology, and growth conditions. Considering the optimization of syntheses and formulation parameters, biocompatibility, biodegradability, and outcomes of the therapeutic applications, several preclinical investigations have been performed in various animals, such as mice (Lim et al., 2009; Liu et al., 2014; Wang et al., 2018; Liang et al., 2022) and rabbit (Liu et al., 2011; Ho et al., 2020; Nilforoushzadeh et al., 2020), to explore their safety and performance efficacy. In most instances, the performance efficacy of these designed carriers have resulted in improved tissue repair and regeneration abilities. However, the performance of microcarriers in a species with long term safety and treatment time considerations yet remain to be elucidated comprehensively. Despite the success, it is still a long way to go to explore the comprehensive evaluations in terms of PK-PD parameters and toxicity attributes. Further, these parameters must be explored and validate their proficiency in humans, requiring extensive investigations and validations.

## 7 Conclusion and perspectives

In conclusion, this article has summarized the discussions on diverse polymeric microarchitectures for minimally-invasive cell delivery towards TE and RM. The significance and classification of diverse injectable microcarriers are initially presented, emphasizing their importance, pros, and cons regarding cell encapsulation and subsequent delivery processes. Further, various microfabrication approaches are explored, stressing their importance in designing cellularized microarchitectures and the feasibility of encapsulating cells and substantial tissue growth *in situ* and *in vivo*.

Recently, several efforts have been dedicated to produce highly biocompatible microcarriers using various biomaterials for cell delivery applications. In this regard, these 3D micro-sized scaffolding systems offered attributes of improved cell encapsulation and delivery efficiencies. Despite the success, several attributes in terms of material fabrication and the performance of delivered cells remain to be addressed. Regarding fabrication, strict optimization of morphological properties (surface and textural attributes) and cell encapsulation are required. Predominantly, the optimal size convenient for injection and the precise evaluation of pore sizes must be explicitly investigated. Regarding cell encapsulation, several steps must be taken to address the fabrication of tissue-like and organ-like multicellular spheroids for TE. Moreover, the encapsulation efficiencies and physiological phenomena, such as apoptosis of encapsulated cells, must be explored to make these 3D microarchitectures more robust. The strict validation of assessments and the establishment of various cell seeding and encapsulation tools is required.

In terms of performance efficacy, these 3D microcarriers carrying cells, after injection, would facilitate the delivery of cells from the exterior to the surrounding ECM. In this view, it is required to explore the pathway of delivered cells in the case of a free-flowing medium. However, it is highly challenging to deliver cells from the interiors due to the excessive growth and proliferation of cells. On the one hand, it is required to explore the controlled proliferation efficiency of cells. On the other hand, the degradation profiling of microcarriers must be explored in case of uncontrolled growth, achieving appropriate physicochemical and mechanical attributes. In addition, control over the cellular microenvironment on the microscale must be achieved as the factors of cell-cell interactions and cell-ECM (integrin and fibronectin) interactions, along with the biochemical cues, play crucial roles. Finally, it is required to explore the functionalities related to integrating with the existing vascularization and neovascularization should be explored along with the validations *in vivo*. Although the fabrication and delivery are achieved to a considerable extent, the reproducibility of these 3D carriers by various microfabrication approaches on a large scale remains to be explored. Along this line, strict optimization of the processing parameters should be done prior to large-scale development.

Despite the success in generating various 3D microarchitectures, the suitability and generation of immune responses would limit the growth of the delivered cells. Interestingly, precision medicine will be the trend soon, in which patient-derived cells can be cultivated and generated for the treatment of individual patients. The success of these models can be achieved by substantially loading the appropriate patient-derived cell lines, providing gradient oxygen and nutrient supply, and considering growth factors. Finally, the optimization on a large scale and their applicability will undoubtedly offer great potential in the future.
